# Supplementary material for: Affinity maturation of antibody responses is mediated by differential plasma cell proliferation
Source: bioRxiv. 2024 Nov 29:2024.11.26.625430. Preprint. [Version 1] doi: 10.1101/2024.11.26.625430 (PMC11623657; doi:10.1101/2024.11.26.625430)
Supplement: 1 [file NIHPP2024.11.26.625430v1-supplement-1.pdf]

bioRxiv preprint doi: https://doi.org/10.1101/2024.11.26.625430; this version posted November 29, 2024. The copyright holder for this preprint (which was not certified by peer review) is the author/funder, who has granted bioRxiv a license to display the preprint in perpetuity. It is made available under aCC-BY 4.0 International license.

Supplementary Figure 1.

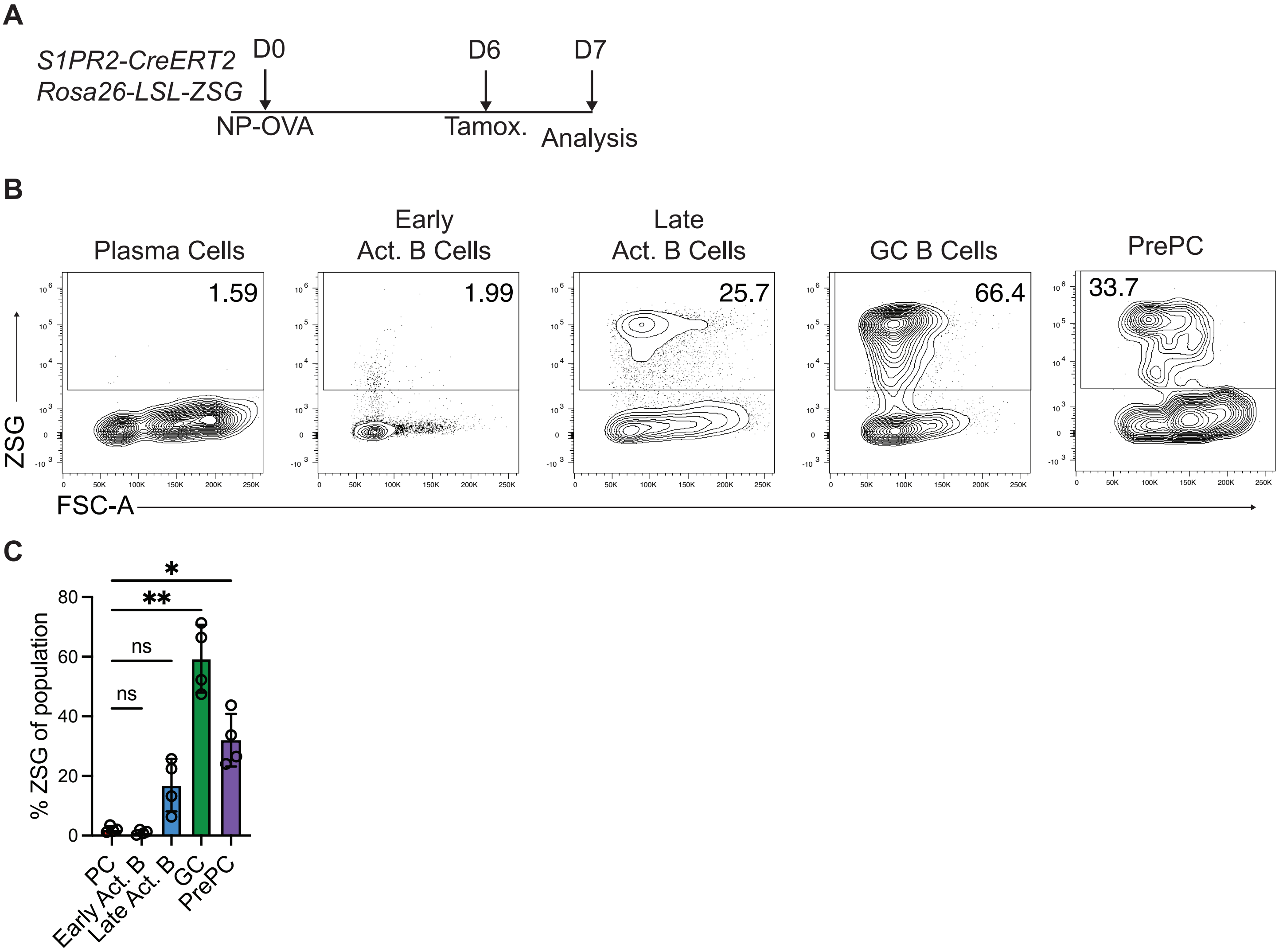

bioRxiv preprint doi: https://doi.org/10.1101/2024.11.26.625430; this version posted November 29, 2024. The copyright holder for this preprint (which was not certified by peer review) is the author/funder, who has granted bioRxiv a license to display the preprint in perpetuity. It is made available under aCC-BY 4.0 International license.

Supplementary Fig. 1. S1PR2-CreERT2 labels GC B cells and late activated B but not mature plasma cells, related to fig.1.

(**A**) Experimental outline. (**B**) Representative flow cytometry plots showing ZSG expression in PCs (TACI^{+} CD138^{+}), early activated B cells (TACI^{-} CD138^{-} B220^{+} CD38^{+} GL7^{+} Fas^{-}), late activated B cells (TACI^{-} CD138^{-} B220^{+} CD38^{+} GL7^{+} Fas^{+}), GC B cells (TACI^{-} CD138^{-} B220^{+} CD38^{-} GL7^{+} Fas^{+}) and PrePC (TACI^{-} B220^{+} CD38^{-} GL7^{+} Fas^{+} CD138^{+}). (**C**) Quantitation of data presented in B. Each point represents one mouse. Data in B-C represent one of three experiments performed. ns, not significant, ** $P<0.005$; ordinary one-way ANOVA.

Supplementary Figure 2.

bioRxiv preprint doi: https://doi.org/10.1101/2024.11.26.625430; this version posted November 29, 2024. The copyright holder for this preprint (which was not certified by peer review) is the author/funder, who has granted bioRxiv a license to display the preprint in perpetuity. It is made available under aCC-BY 4.0 International license.

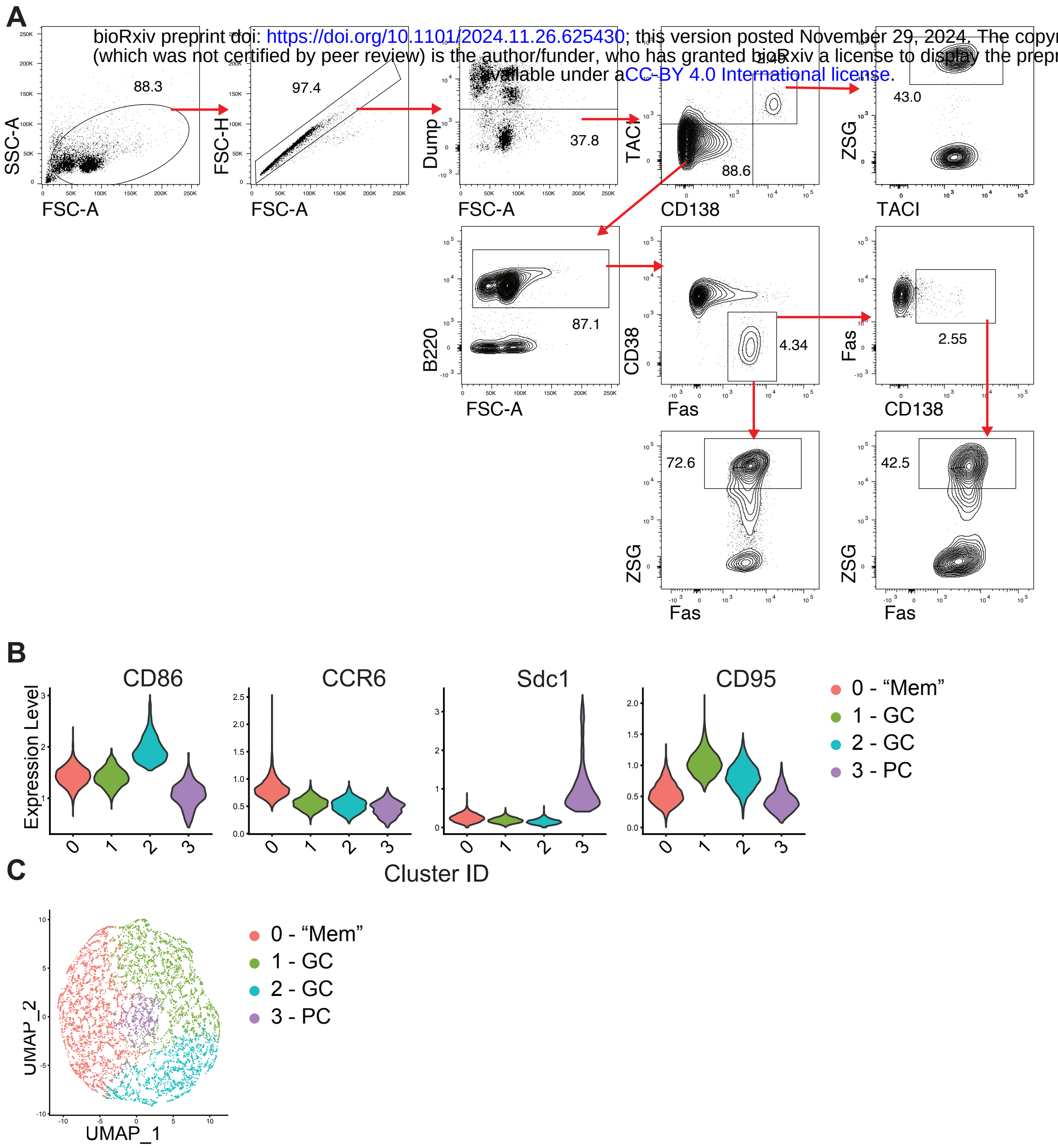

bioRxiv preprint doi: https://doi.org/10.1101/2024.11.26.625430; this version posted November 29, 2024. The copyright holder for this preprint (which was not certified by peer review) is the author/funder, who has granted bioRxiv a license to display the preprint in perpetuity. It is made available under aCC-BY 4.0 International license.

Supplementary Fig. 2. Identification of GC B and PC, related to fig.1

(**A**) Gating strategy for ZSG^{+} PCs ($Dump^{-}$ $CD138^{+}$ $TACI^{+}$), GC B ($Dump^{-}$ $TACI^{-}$ $B220^{+}$ $CD38^{lo}$ Fas^{+}) and prePCs ($Dump^{-}$ $TACI^{-}$ $B220^{+}$ $CD38^{lo}$ Fas^{+} $CD138^{+}$), from animals treated as in Fig. 1A. Gating approach depicted was used for these populations throughout unless otherwise stated. (**B**) Expression levels of CITE-seq surface staining in clusters identified as PC and GC in two separate sequencing runs. (**C**) Uniform manifold approximation and projection (UMAP) of the above data. Data are pooled from 5 mice, and represent two experiments performed.

bioRxiv preprint doi: https://doi.org/10.1101/2024.11.26.625430; this version posted November 29, 2024. The copyright holder for this preprint (which was not certified by peer review) is the author/funder, who has granted bioRxiv a license to display the preprint in perpetuity. It is made available under aCC-BY 4.0 International license.

Supplementary Figure 5

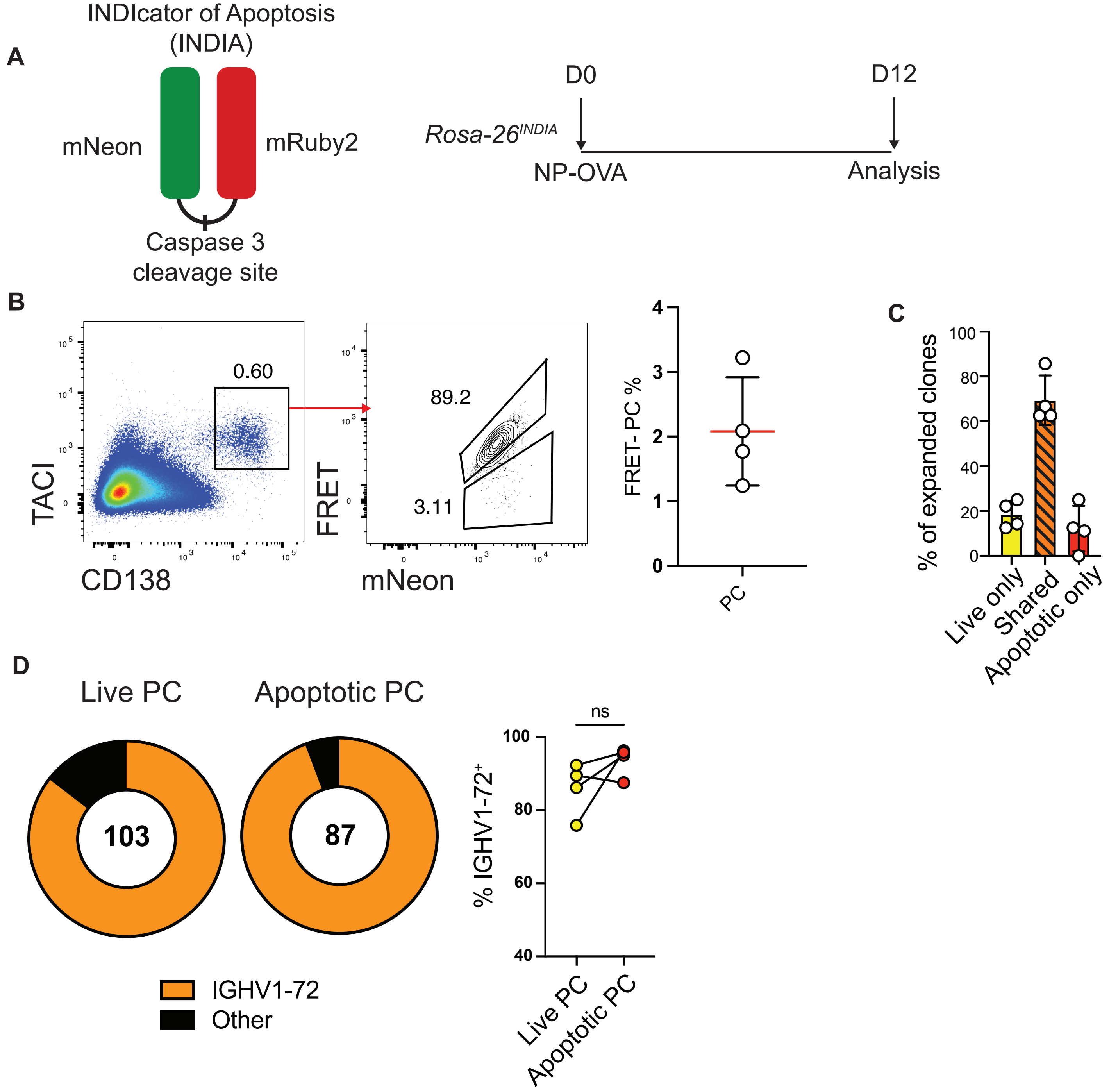

bioRxiv preprint doi: https://doi.org/10.1101/2024.11.26.625430; this version posted November 29, 2024. The copyright holder for this preprint (which was not certified by peer review) is the author/funder, who has granted bioRxiv a license to display the preprint in perpetuity. It is made available under aCC-BY 4.0 International license.

Supplementary Fig. 3. Plasma cell apoptosis is not associated with BCR affinity.

(**A**) Left, Diagram of INDIA reporter. Right, Experimental outline for (**B-D**). (**B**) Left, representative flow cytometry plot showing gating for $CD138^{+}TACI^{+}$ PCs, FRET (BB630 channel) and mNeon. Right, Quantitation of $FRET^{+}$ PCs. (**C**) Frequency of clones found only among live PCs, only in $FRET^{-}$ apoptotic PCs or 'shared' clones found in both populations. Each point represents one mouse. (**D**) Left, frequency of $FRET^{+}$ live PCs or $FRET^{-}$ apoptotic PCs expressing IGHV1-72 antibodies. Right, summary of IGHV1-72 frequencies, each point represents one mouse. All experiments were performed at least twice.

bioRxiv preprint doi: https://doi.org/10.1101/2024.11.26.625430; this version posted November 29, 2024. The copyright holder for this preprint (which was not certified by peer review) is the author/funder, who has granted bioRxiv a license to display the preprint in perpetuity. It is made available under aCC-BY 4.0 International license.

Supplementary Figure 4.

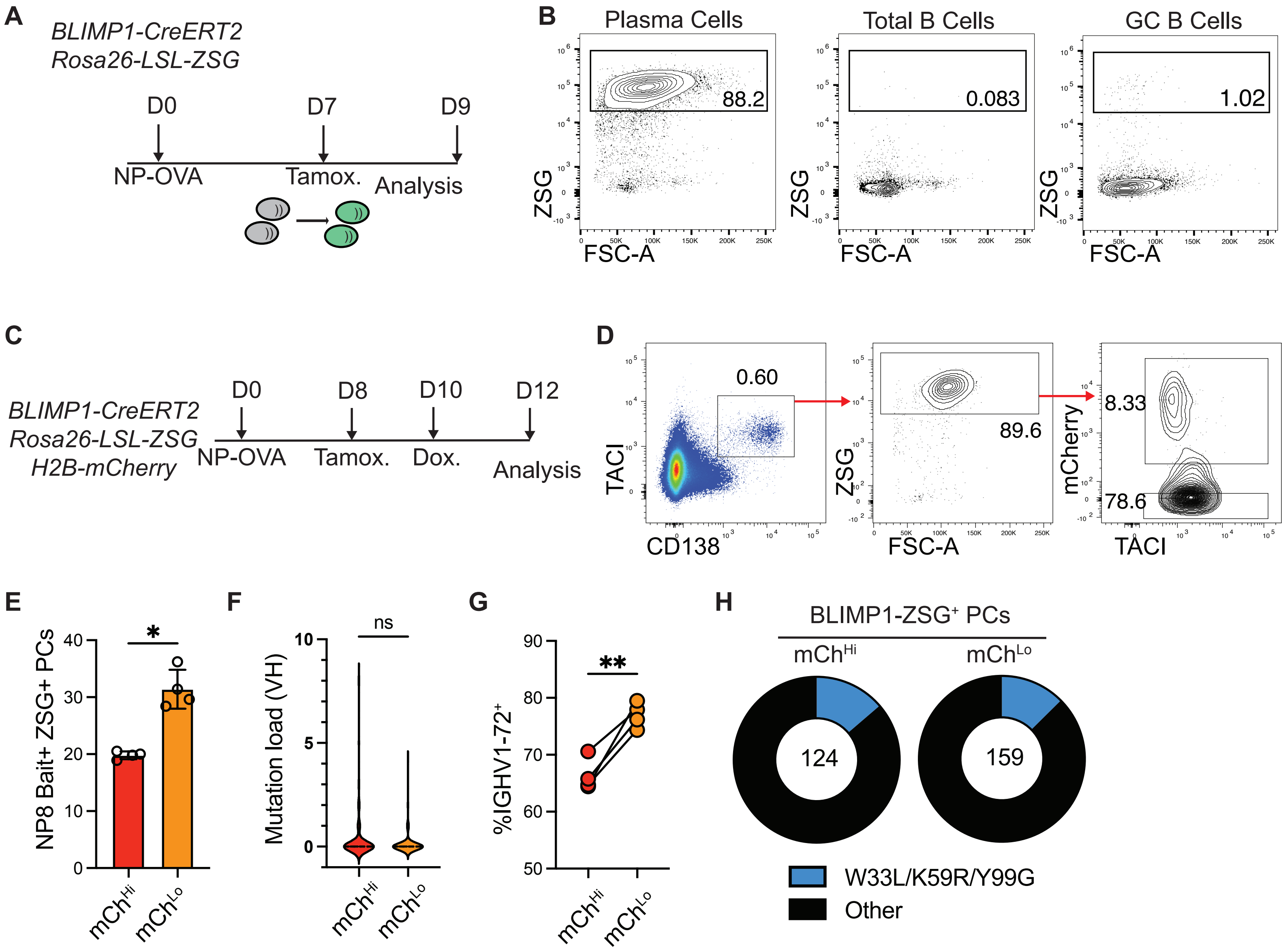

bioRxiv preprint doi: https://doi.org/10.1101/2024.11.26.625430; this version posted November 29, 2024. The copyright holder for this preprint (which was not certified by peer review) is the author/funder, who has granted bioRxiv a license to display the preprint in perpetuity. It is made available under aCC-BY 4.0 International license.

Supplementary Fig. 4. Proliferating PCs are enriched among high-affinity antigen binding cells. (**A**) Experimental layout for **B**. (**B**) Representative flow cytometry plots showing Blimp1-CreERT2-driven fate mapping of PCs ($CD138^{+}TACI^{+}$), total B cells ($TACI^{-}$ $CD138^{-}$ $B220^{+}$) and GC B cells ($TACI^{-}$ $CD138^{-}$ $B220^{+}CD38^{-}Fas^{+}$). (**C**) Experimental layout for **D-H**. (**D**) Flow cytometry profile showing $TACI^{+}CD138^{+}ZSG^{+}$ PCs and gating for mCh^{hi} and mCh^{lo} cells from pLNs. (**E**) Quantitation of NP bait staining frequency among mCh^{hi} and mCh^{lo} PCs on D12 after immunization. (**F**) Number of VH mutations in ZSG^{+} PC populations.(**G**) Frequency of mCh^{Hi} or mCh^{Lo} ZSG^{+} PCs expressing IGHV1-72. (**H**) Frequency of high affinity mutation containing sequences among IGHV1-72^{+} expressing mCh^{Hi} or mCh^{Lo} ZSG^{+} PCs. ns, not significant * $p<0.05$, ** $p<0.005$. (**E, G**) paired two-tailed Student's t-test; (**F**) unpaired Student's t-test. Data are pooled from 2 independent experiments, n=4.

bioRxiv preprint doi: https://doi.org/10.1101/2024.11.26.625430; this version posted November 29, 2024. The copyright holder for this preprint (which was not certified by peer review) is the author/funder, who has granted bioRxiv a license to display the preprint in perpetuity. It is made available under aCC-BY 4.0 International license.

Supplementary Figure 5

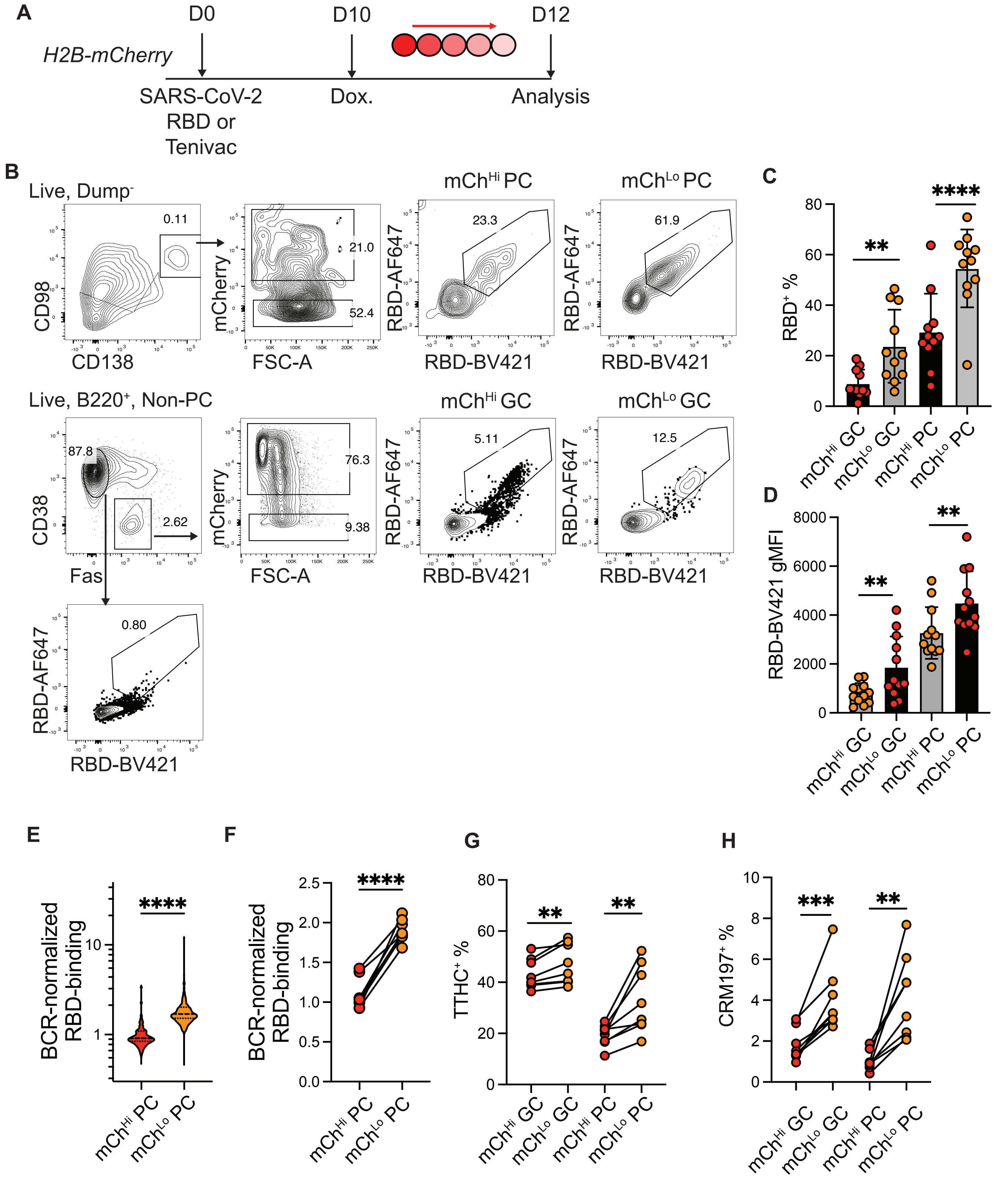

bioRxiv preprint doi: https://doi.org/10.1101/2024.11.26.625430; this version posted November 29, 2024. The copyright holder for this preprint (which was not certified by peer review) is the author/funder, who has granted bioRxiv a license to display the preprint in perpetuity. It is made available under aCC-BY 4.0 International license.

Supplementary Fig. 5. Proliferating PCs are enriched among high-affinity antigen binding cells. (**A**) Experimental layout used in (**B-D**). (**B**) Representative flow cytometry plots showing gating on mCh^{hi} and mCh^{lo} $CD98^{+}CD138^{+}$ PCs, and $mCh^{hi/lo}$ $B220^{+}CD38^{-}Fas^{+}$ GC B cells. Right panels display representative dual antigen bait staining for Sars-CoV-2 RBD. Naïve $B220^{+}CD38^{+}Fas^{-}$ B cells were used as a negative control for bait staining. (**C**) Quantitation of RBD staining frequency among mCh^{hi} and mCh^{lo} GC B cells and PCs on D12 after SARS-CoV-2 RBD immunization. (**D**) Geometric mean fluorescence intensity (gMFI) of RBD-BV421 staining among mCh^{hi} and mCh^{lo} PC and GC B. (**E**) Violin plots displaying cellular distribution of BCR-normalized bait binding. (**F**) Average BCR-normalized bait binding. Each point represents one mouse. (**G-H**) Quantitation of tetanus toxoid heavy chain fragment c (TTHC; **E**) and detoxified diphtheria toxin (CRM197; **F**) bait binding on D12 after Tenivac immunization. * $p<0.05$, ** $p<0.005$, ***$p<0.0005$, **** $p<0.0001$. (**C-D, F-H**) Paired two-tailed Student's t-tests. (**E**), Mann-Whitney test. Data in B-F and G-H are each representative of 3 independent experiments.

bioRxiv preprint doi: https://doi.org/10.1101/2024.11.26.625430; this version posted November 29, 2024. The copyright holder for this preprint (which was not certified by peer review) is the author/funder, who has granted bioRxiv a license to display the preprint in perpetuity. It is made available under aCC-BY 4.0 International license.

Supplementary Figure 6

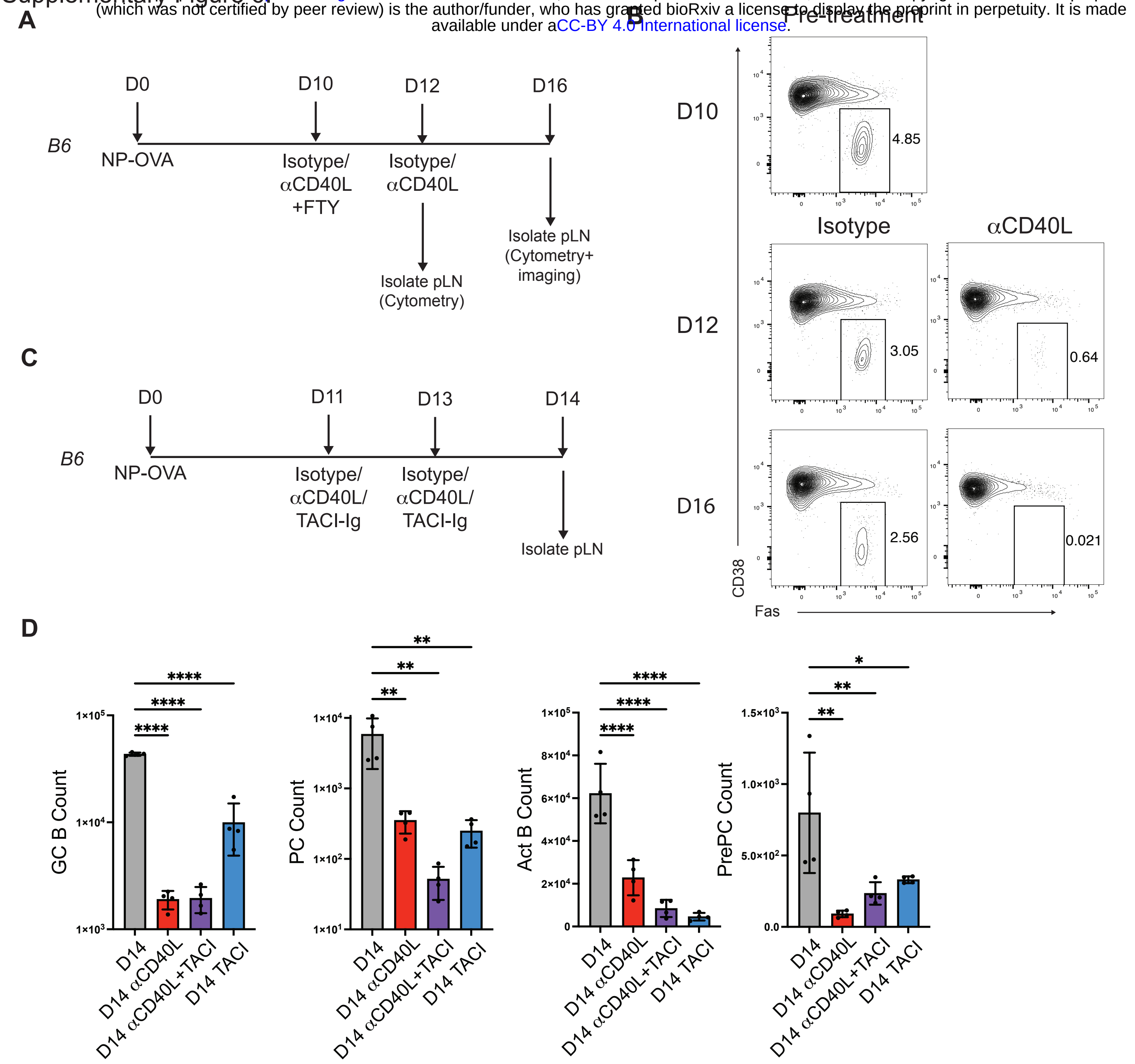

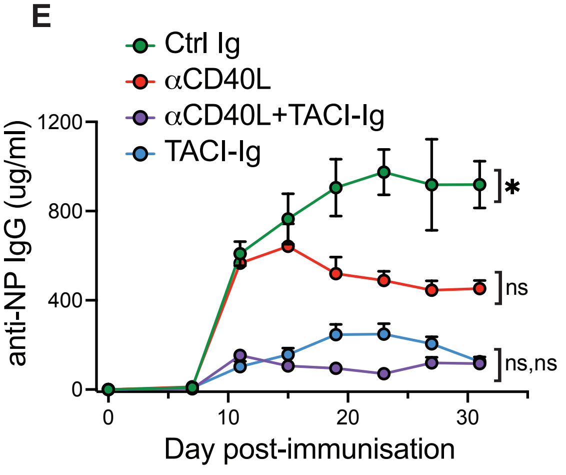

F

	Treatment group			
Comparison	Ctrl (Isotype)	αCD40L	TACI-Ig	αCD40L+ TACI-Ig
D15 vs D11	ns	ns	ns	ns
D19 vs D11	**	***	ns	ns
D23 vs D11	****	**	ns	ns
D27 vs D11	****	**	ns	ns
D32 vs D11	***	*	ns	ns

bioRxiv preprint doi: https://doi.org/10.1101/2024.11.26.625430; this version posted November 29, 2024. The copyright holder for this preprint (which was not certified by peer review) is the author/funder, who has granted bioRxiv a license to display the preprint in perpetuity. It is made available under aCC-BY 4.0 International license.

Supplementary Fig. 6, related to Fig. 3 and Fig.4. GC B and PC depletion kinetics.

(**A**) Experimental layout used in (**B**). (**B**) Representative cytometry plots showing GC B cell depletion in pLNs on D12 and D16 after immunization. (**C**) Experimental layout used in (**D**). (**D**) Quantitation of GC B, PC, activated B and prePCs after treatment with depleting antibodies as described in (**C**). (**E**) Total serum NP-binding IgG, from mice treated as in Fig. 4H, as measured by NP_{28}-binding. Statistical comparisons shown represent results of a mixed-effects analysis, from endpoint D32 vs D11 onset of treatment. (**F**) Results of mixed effects analysis comparing affinity maturation (NP_7/NP_{28} ratio) of the specified timepoints vs D11, in the same group. Data are presented in Fig.4D,E. * $p<0.05$, ** $p<0.005$, ***$p<0.0005$, **** $p<0.0001$, ns not significant. (**D**) Ordinary one-way ANOVA (all plots); (**E,F**) mixed-effects analysis.

bioRxiv preprint doi: https://doi.org/10.1101/2024.11.26.625430; this version posted November 29, 2024. The copyright holder for this preprint (which was not certified by peer review) is the author/funder, who has granted bioRxiv a license to display the preprint in perpetuity. It is made available under aCC-BY 4.0 International license.

Supplementary Figure 7.

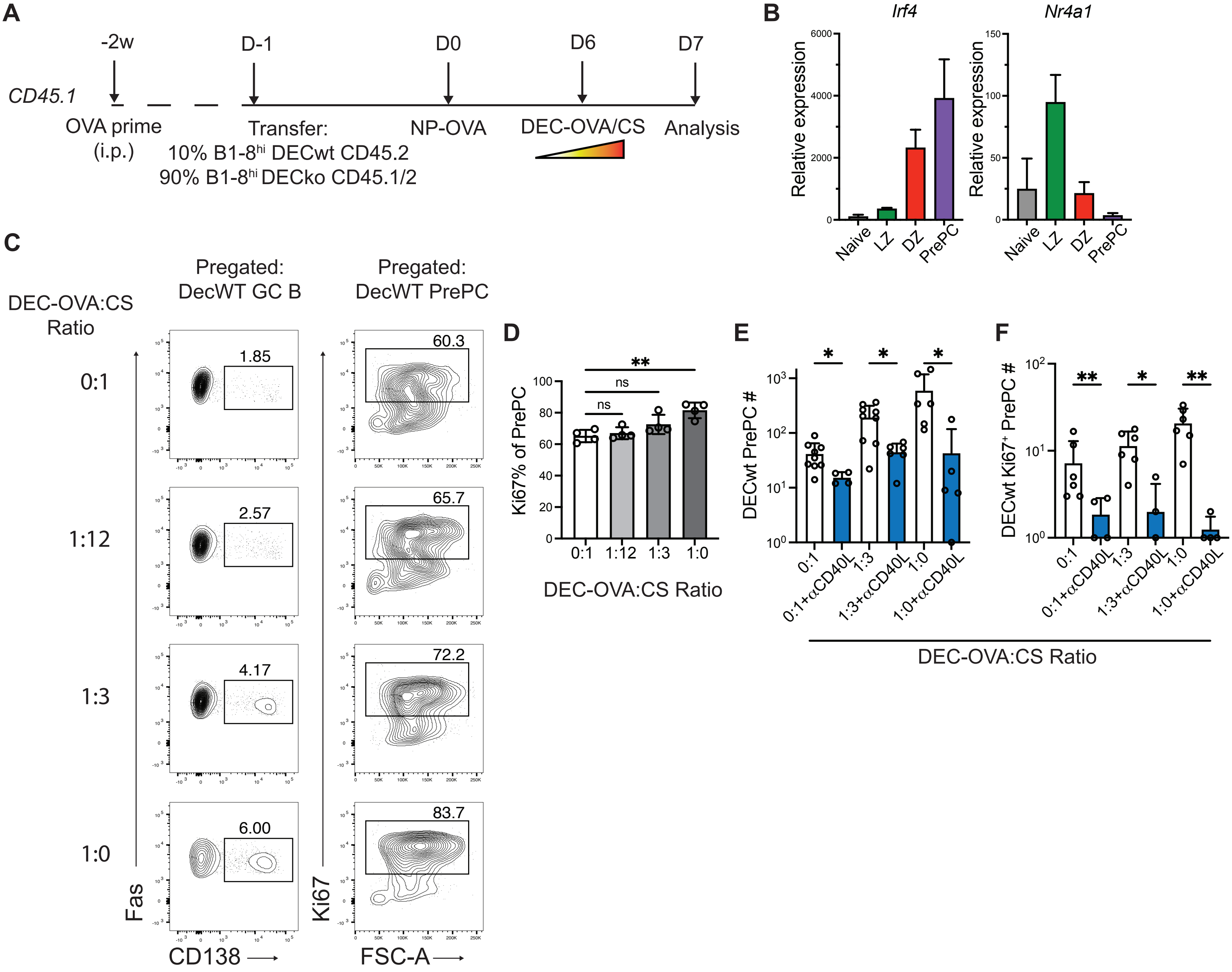

bioRxiv preprint doi: https://doi.org/10.1101/2024.11.26.625430; this version posted November 29, 2024. The copyright holder for this preprint (which was not certified by peer review) is the author/funder, who has granted bioRxiv a license to display the preprint in perpetuity. It is made available under aCC-BY 4.0 International license.

Supplementary Fig 7. PrePC response to T cell help.

(**A**) Experimental layout used in (**B-D**). (**B**) qPCR of purified naïve B (grey bars) LZ B (green bars), DZ B (red bars) and prePC (purple bars) showing GAPDH-normalized relative expression for *Irf4* (left) and *Nr4a1* (right). (**C**) Representative cytometry plots showing frequency of prePC and Ki67 staining among prePC, after DEC-OVA:DEC-CS administration. (**D**) Percentage of $Ki67^{+}$ cells among total DEC^{WT} prePC. (**E-F**) Quantitation of DEC^{WT} prePCs (**E**) and $Ki67^{+}$ DEC^{WT} prePCs (**F**) 72h after anti-DEC administration, with or without aCD40L treatment as indicated (also see Fig.5G). * p<0.05, ** p<0.005. (**D-F**) Kruskal-Wallis tests.

bioRxiv preprint doi: https://doi.org/10.1101/2024.11.26.625430; this version posted November 29, 2024. The copyright holder for this preprint (which was not certified by peer review) is the author/funder, who has granted bioRxiv a license to display the preprint in perpetuity. It is made available under aCC-BY 4.0 International license.

Supplementary Figure 8.

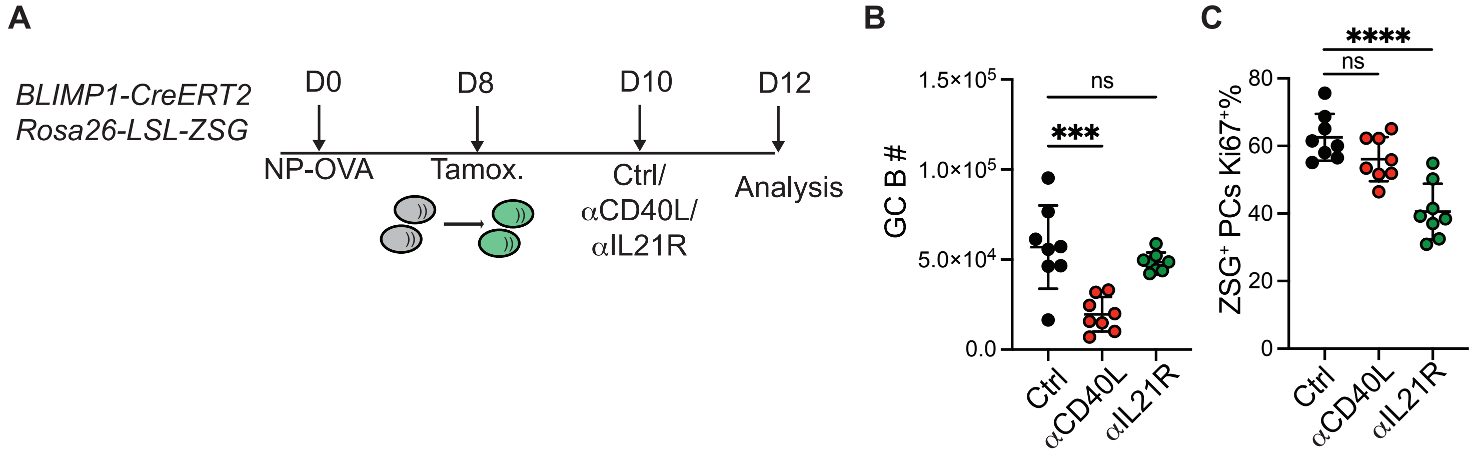

bioRxiv preprint doi: https://doi.org/10.1101/2024.11.26.625430; this version posted November 29, 2024. The copyright holder for this preprint (which was not certified by peer review) is the author/funder, who has granted bioRxiv a license to display the preprint in perpetuity. It is made available under aCC-BY 4.0 International license.

Supplementary Fig 8. IL-21R supports post-GC expansion of PCs. (**A**) Experimental layout. (**B**) GC B cell numbers from mice treated with aCD40L, aIL-21R or isotype control antibodies between D10-D12. (**C**) Frequency of Ki67+ dividing cells amongst CD138^{+} TACI^{+} ZSG^{+} fate-mapped PCs. ns, not significant ** $p<0.005$; (**B**,**C**) Ordinary one-way ANOVA. Data are pooled from 3 independent experiments.
